# Custom-Made Direct Metal Laser Sintering Titanium Subperiosteal Implants: A Retrospective Clinical Study on 70 Patients

**DOI:** 10.1155/2018/5420391

**Published:** 2018-05-28

**Authors:** Mauro Cerea, Giorgio Andrea Dolcini

**Affiliations:** ^1^Private Practice, 24128 Bergamo, Italy; ^2^Private Practice, 21100 Como, Italy

## Abstract

**Purpose:**

To present a digital technique for the fabrication of custom-made subperiosteal implants and to report on the survival and complication rates encountered when using these fixtures.

**Methods:**

The data used for this retrospective clinical study were derived from the medical records of five different private dental practices. Inclusion criteria were patients over the age of 60, treated with custom-made direct metal laser sintering (DMLS) titanium subperiosteal implants (Eagle-Grid®, BTK, Dueville, Vicenza) during a two-year period (2014-2015) and restored with fixed restorations; all enrolled patients needed to have complete pre- and postoperative clinical and radiographic documentation, with at least 2 years of follow-up. Exclusion criteria were smoking and bruxism. The main outcomes looked at were implant survival and complications.

**Results:**

Seventy patients (39 males and 31 females, aged 62-79 years) who had been treated with custom-made DMLS titanium subperiosteal implants were enrolled in this study. After 2 years of follow-up, three implants were lost due to recurrent, untreatable infections; the survival rate was therefore 95.8% (67/70 implants). Four patients reported pain/discomfort/swelling after implant placement; the incidence of immediate postoperative complications was therefore 5.7% (4/70 implants). During the follow-up period, one patient suffered from recurrent infections classified as a biologic complication; the incidence of biologic complications was therefore 1.4% (1/67 surviving implants). Finally, four patients experienced prosthetic problems with their implant-supported restorations during the provisional phase (fracture of the acrylic restoration) and two patients had ceramic chipping of the definitive restoration; the incidence of prosthetic complications was therefore 8.9% (6/67 surviving implants).

**Conclusions:**

Within the limits of the present study (limited follow-up time and low number of patients treated, retrospective design), the application of custom-made DMLS titanium subperiosteal implants showed satisfactory implant survival (95.8%) and low complication rates. Further studies are needed to confirm the positive outcomes found in this research.

## 1. Introduction

Endosseous dental implants provide a highly predictable solution for the prosthetic rehabilitation of partially and totally edentulous patients, with high rates of survival and success in the medium and long terms [1–3].

An adequate quantity (height and width) and quality (density) of bone are needed to be able to place endosseous dental implants [4, 5].

Cases may occur, however, of patients with severe bone atrophy, for whom the placement of endosseous dental implants may be impossible without the use of regenerative surgical techniques [4, 5].

Several surgical techniques have been proposed for bone regeneration to allow for the subsequent placement of endosseous implants; these include onlay/inlay bone grating [6, 7], guided bone regeneration (GBR) with resorbable or nonresorbable membranes [8], alveolar ridge split [9], distraction osteogenesis [10], and maxillary sinus augmentation [11, 12].

All of these regenerative techniques, which make use of different materials (autogenous bone harvested from intraoral/extraoral sites; homologous, heterologous, or synthetic bone grafts) may restore bone volume to a level that allows the proper placement of endosseous dental implants [6–14]; however, these surgical techniques are complex and can have a rather high percentage of complications [5, 6, 10, 13, 15]. In addition, they lengthen the time of treatment and present additional costs to the patient [5, 6, 10, 13, 15].

Different surgical techniques for the placement of endosseous dental implants in an unfavourable anatomical situation (i.e., without bone regeneration) have been proposed: the use of short [16], narrow [17], or tilted implants [18] or the use of zygoma [19] or pterygomaxillary implants [20]. The former three procedures have been very successful in modern implantology and are increasingly used [16–18], while the latter two remain niche treatments [19, 20]. In any case, a minimum level of endosseous bone volume is needed to place short, narrow, or tilted endosseous implants; in cases of severe or advanced bone atrophy, the use of such nonregenerative surgical approaches may not be possible [16–18].

In the past, and before they were abandoned in favour of endosseous implants, there was the possibility of inserting subperiosteal implants in cases of severe bone atrophy [21–23].

A subperiosteal implant is a type of dental implant that is placed between the periosteum and the residual alveolar bone [21–23]. It usually has two to four transmucosal elements projecting through the mucosa into the oral cavity, connecting the implant to the prosthesis [21–23].

Introduced in the early 1940s in Sweden [21, 24] and then in the United States [24], subperiosteal implants were traditionally made from chrome-cobalt or titanium alloys [24–27]. They were usually placed above the bone, in cases of severe bone resorption of the ridges, and were often immediately loaded with a removable or fixed prosthesis [22, 24, 28].

Although such implants enjoyed a certain popularity and level of success for a period of about twenty years [24–30], they were subsequently replaced by endosseous implants, as proposed by Branemark [4, 31]. This change occurred for several reasons [32]. First, the fabrication technique was complex: it required taking a physical impression of the residual bone anatomy, resulting in considerable discomfort for the patient (due to the need for a rather large skeletonization at the stage of impression taking) [4, 21, 23–30]. Direct bone impressions led to the fabrication of a framework/mesh in the laboratory [4, 21, 23–30]. This traditional fabrication technique could result in a nonideal adaptation to the recipient bone site; the positioning technique was complex and time-consuming and led to a higher risk of postoperative infections and complications [4, 28, 30, 33–35].

Today, however, the world of dentistry has changed, thanks to the powerful impact of the digital revolution [36]. The introduction of new acquisition methods (cone beam computed tomography (CBCT) [37] and intraoral scanners [38]), along with the spread of processing/elaboration such as computer-assisted-design/computer-assisted-manufacturing (CAD/CAM) software [39], new materials [40], and fabrication technologies, has dramatically changed the world of implant dentistry, opening up new perspectives.

In particular, the new direct metal laser sintering (DMLS) techniques available today provide the ability to fabricate custom-made grids [41] or even implants [42, 43] that perfectly adapt to the specific anatomical requirements of patients. The use of such modern fabrication techniques allows us to revisit some of the past techniques, such as subperiosteal implants, and reinterpret them in a modern and digital way [44]. This can be extremely useful in the case of severe bone atrophy, which does not allow for the placement of endosseous dental implants if a regenerative intervention approach is not followed, particularly in the case of elderly patients with limited financial resources and who do not wish to undergo long and complex regenerative surgeries prior to the insertion of endosseous dental implants [44].

The purpose of the present retrospective clinical study is to present a new digital technique for the fabrication of custom-made DMLS subperiosteal implants and to report on the survival and complication rates encountered when using these fixtures.

## 2. Methods

### 2.1. Patient Selection

The data for this retrospective clinical study were derived from the electronic medical records of the private dental practice of Dr. Mauro Cerea, as well as from the clinical records of four private dental practices, located in the north of Italy, in which Dr. Cerea worked as an oral and maxillofacial surgeon, over a two-year period (January 2014 to June 2015). These clinical records contained presurgical documentation of cases and complete surgical and postsurgical documentation, including information about any failures or complications that occurred during intervention, healing period, and follow-up.

The inclusion criteria for enrolment in the study were patients over the age of 60, treated with custom-made DMLS titanium subperiosteal implants during the period from January 2014 to June 2015, and restored with fixed restorations; all enrolled patients had to present complete pre- and postoperative clinical and radiographic documentation, with at least 2 years of follow-up from implant placement. All patients had been asked to attend a 2-year follow-up visit, and attendance was an inclusion criterion for enrolment in this study.

All patients who did not attend the 2-year follow-up examination, as well as all cases lacking complete clinical and radiological documentation with at least 2 years of follow-up, were excluded from the study; smokers and bruxist patients were also excluded.

This retrospective study was conducted in accordance with the principles set forth in the Helsinki Declaration on Human Subject Experimentation (2008 revision).

### 2.2. Preoperative Workup and CBCT Examination

All patients were initially subjected to two-dimensional radiographic evaluation via orthopanoramic (OPT) radiography. In addition, accurate impressions of the partially/totally edentulous arches were taken and a diagnostic wax-up was performed in order to better understand the prosthetic needs of the patients. The diagnostic wax-up provided the shape/dimensions and the teeth of the final prosthesis that would eventually be cemented onto the abutments integrated into the subperiosteal implant structure. If the patient carried a complete removable prosthesis, the removable prosthesis was duplicated to create the diagnostic wax-up. The dental technician then prepared a resin scan prosthesis, a copy of the diagnostic wax-up. The patient needed to wear this scan prosthesis, with mouth closed and arches in occlusion, during the execution of cone beam computed tomography (CBCT) for the three-dimensional (3D) evaluation of residual bone anatomy (size and shape of the residual bone, height, thickness, and angulation). Finally, the resin scan prosthesis and the patients' plaster models were scanned, independently and together, with a desktop scanner, and saved as STL files.

### 2.3. Design and Fabrication of the Custom-Made Subperiosteal Implants

The DICOM data derived from the CBCT were then imported into a bone reconstruction software program (3D Slicer®, BTK-3D, Dueville, Vicenza) in which an accurate 3D reconstruction of the patient's remaining bone was performed using a thresholding procedure. At the end of this procedure, the virtual reconstruction of the patient's bone anatomy was saved in STL format. This file was then aligned with the STL files of the patient's plaster model and the diagnostic wax-up, in order to obtain a complete, virtual model of the patient, with all information about bone, teeth, soft tissues, and the new prosthesis. This STL file was then processed and printed, in resin, using a powerful rapid prototyping machine (ProJet 3510 MP®, 3D Systems, Rock Hill, SC, USA). The accuracy of this resin model mainly depended on the type and resolution of acquisition of the CBCT; a smaller field of view (FOV) was therefore selected when possible (for example, in the case of partially edentulous patients) in order to provide better resolution for the acquisition. This resin model was only produced to test the accuracy and fit of the subperiosteal implant after fabrication via direct metal laser sintering (DMLS). The patient's 3D virtual bone model was then imported into a proprietary modeling/processing software program (PlastyCAD®, BTK-3D, Povolaro di Dueville, Vicenza), where the subperiosteal implant was designed virtually, following the instructions of the clinician. The customized implant already included the holes for the fixing screws and the integral abutments for the support of the cemented fixed prosthetic rehabilitation. Again, the virtual model of the subperiosteal implants was saved as an STL file; this file was first printed in resin to verify the accuracy of the fit on the 3D printed model of the patient. After the accuracy was checked and verified, the custom-made subperiosteal implant was sent to a powerful direct metal laser sintering (DMLS) machine (ProX-DMP100®, 3D Systems, Rock Hill, SC, USA) for fabrication. The customized, anatomically shaped implant was fabricated layer by layer, directly from grade 5 titanium micropowders. The DMLS process created a porous, chemically pure implant from 3D CAD data by melting fine titanium powders with a laser beam (50/W fibre laser with a wavelength of 1070 nm), layer by layer (with layer sizes ranging from 10 to 20 *μ*m). The build envelope capacity of the machine was 100 × 100 × 80 mm. Once produced, the implant was polished through electroerosion. After polishing, it was decontaminated in a clean room and packaged for sterilization. The dental lab then proceeded to manufacture the CAD/CAM fixed implant-supported prostheses for both partially and totally edentulous patients.

### 2.4. Surgical and Prosthetic Procedures

After local anaesthesia was performed by infiltration with 4% articaine containing 1:100,000 adrenaline, a wide crestal incision was made in the edentulous area, connected by two deep releasing incisions, mesially and distally. Subsequently, a full-thickness flap was raised to make the bone tissue clearly visible where the custom-made implant was to be placed. The custom-made implant (Eagle-Grid®, BTK, Dueville, Vicenza) was removed from its sterile packaging and its fit on the residual bone was carefully checked; in the case of small undercuts, perfect adaptation of the grid was obtained through light percussion on the integrated abutments. After the position and fit of the grid were checked, the surgeon fixed the implant onto the receiving bone site via placement of different osteosynthetic miniscrews. Finally, the flaps were repositioned and sutures were performed to obtain a tension-free, first-intention closure. A series of releasing periosteal incisions were also performed to better mobilize the flap. Care was taken to obtain a good emergence profile of the abutments, which were surrounded by keratinized gingiva. Following the procedure, the patient was prescribed oral antibiotics, amoxicillin plus clavulanic acid (Augmentin®; GlaxoSmithKline Beecham, Brentford, UK), 1 g every 12 hours for 6 days total, and painkillers, 600 mg ibuprofen (Brufen®, Abbott srl, Chicago, IL, USA), 600 mg/day for 2-3 days. The patient was directed to rinse with 0.12% chlorhexidine mouth rinse (Chlorhexidine®, Oral-B, Boston, MA, USA) 2-3 times a day for 5 days.

**Figure 1:**
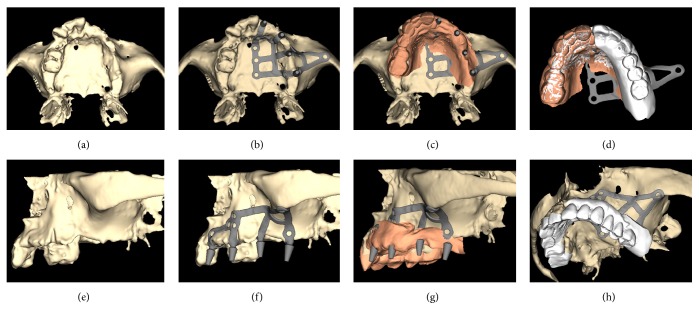
Presurgical planning and modeling of the Eagle-Grid®: (a) 3D bone reconstruction, occlusal view; (b) the subperiosteal implant and its integral abutments in position, occlusal view; (c) overlapping of the soft tissues for the evaluation of the emergency profiles of the prosthetic abutments, occlusal view; (d) preliminary evaluation of the possible prosthetic reconstruction, occlusal view; (e) 3D bone reconstruction, lateral view; (f) the subperiosteal implant and its integral abutments in position, lateral view; (g) overlapping of the soft tissues for the evaluation of the emergency profiles of the prosthetic abutments, lateral view; (h) preliminary evaluation of the possible prosthetic reconstruction, lateral view.

**Figure 2:**
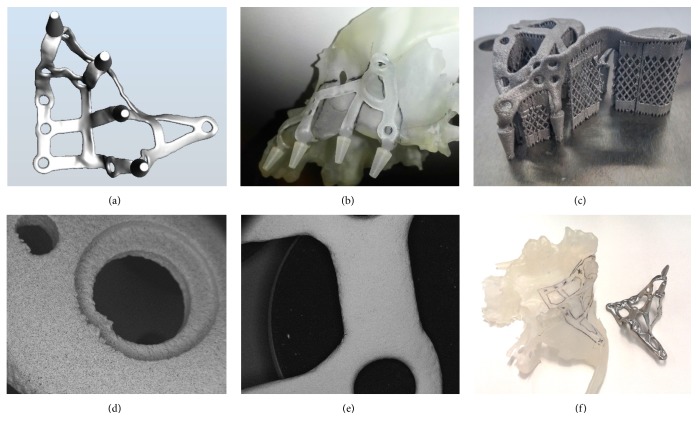
Fabrication of the subperiosteal implant: (a) design of the implant; (b) prior to DMLS, the implant is fabricated in resin, with stereolithography, for better evaluation of the case, directly on the 3D printed model of the bone; (c) the implant is fabricated with DMLS; (d) scanning electron microscopy (SEM) image of the implant surface before the electroerosion treatment (x43); (e) scanning electron microscopy (SEM) image of the implant surface after the electroerosion treatment (x26); (f) the final aspect of the implant that is placed on the 3D printed model of the bone.

**Figure 3:**
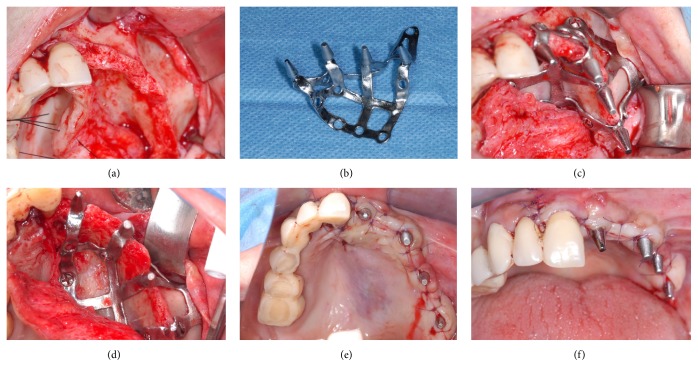
Surgery: (a) the residual bone anatomy; (b) the custom-made DMLS titanium implant; (c) application of the implant; (d) the implant is stabilized via the fixation of osteosynthesis screws; (e) sutures, occlusal view; (f) sutures, frontal view.

**Figure 4:**
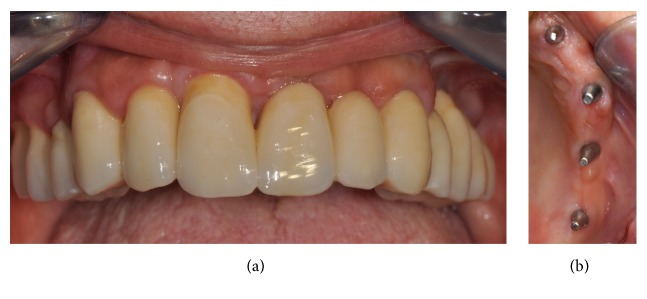
Application of the final prosthetic restoration: (a) frontal view; (b) soft tissues appearance, occlusal view.

**Figure 5:**
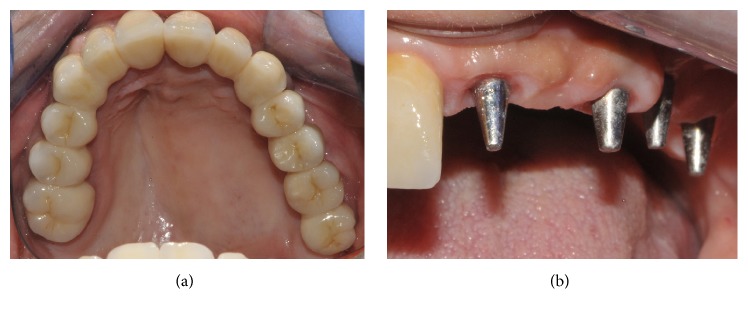
The 2-year control: (a) the prosthetic restoration in position, occlusal view; (b) soft tissues appearance, lateral view.

Within 48 hours, the temporary fixed restoration in acrylic resin, made with CAD/CAM procedures, was delivered to the patient, relined on the abutments, and cemented with a temporary cement; the impression for the fabrication of the final restoration was scheduled to be taken 3-4 months after surgery, with polyvinylsiloxane. The final metal-ceramic restoration was then delivered to the patient within 1-2 weeks and cemented with temporary cement above the titanium abutments. A complete clinical case is documented in Figures 1–5.

### 2.5. Clinical Outcomes

The main outcomes measured in the present study are as follows.

(i) Implant survival: an implant was considered to be “surviving” if still functioning at the end of the 2-year follow-up period; conversely, all implants that suffered from major biologic or prosthetic complications and that needed to be removed were considered “failed.”

(ii) Postoperative complications: biologic complications that occurred immediately after implant placement, such as pain, swelling, oedema, bleeding, or any other problems related to the surgical act.

(iii) Any biologic or prosthetic complications that occurred during the 2-year follow-up period. Biologic complications included severe and/or recurrent implant infections, with exudation/suppuration, pain, swelling, and pus formation, with or without radiographic evidence of bone loss. Prosthetic complications included complications such as fractures of the implant or implant abutments and technical complications affecting the provisional implant-supported fixed restoration (e.g., acrylic resin fractures) or definitive implant-supported rehabilitation (e.g., fractures of the metallic framework, ceramic chipping/fractures).

### 2.6. Statistical Analysis

At the end of the study, all relevant patient data (gender, age at surgery) and implant and prosthesis information, including implant failure and postoperative and biologic/prosthetic complications, were gathered in an Excel spreadsheet. Means (± SD), ranges, medians, and confidence intervals (CI 95%) were calculated for quantitative variables (patients' age), while absolute and relative (%) frequency distributions were obtained for all qualitative variables (patient's gender, complications). Implant survival was calculated at the patient level.

## 3. Results

In total, 75 patients who had received a custom-made DMLS titanium subperiosteal implant during the period between January 2014 and June 2015 were considered for enrolment in the present study.

Among those, three did not have complete clinical and radiographic documentation (two patients were hospitalized for severe illness not related to the implant treatment, and one patient moved to another city), and two did not attend the final 2-year clinical follow-up visit. These five patients were excluded from the study.

The final group thus included 70 patients (39 males and 31 females, aged between 62 and 79 years, mean age 67.8 years, SD 4.1, median 67, CI 95% 66.9-68.7) who were treated with a custom-made DMLS titanium subperiosteal implant in the period between January 2014 and June 2015.

At the end of the study, only three implants were lost, due to recurrent, untreatable infections. These infections forced the surgeon to remove the implants. The other 67 implants (67/70) were functioning normally at the 2-year follow-up visit, giving an overall implant survival rate of 95.8%.

With regard to immediate postoperative complications, four patients reported pain/discomfort after implant placement, with some swelling and bleeding. However, these phenomena resolved within a few days following treatment by analgesics and antibiotics. The incidence of immediate postoperative complications was therefore 5.7% (4/70 implants).

Finally, with regard to biologic and prosthetic complications during the follow-up period, one patient suffered from recurrent infections that were classified as a biologic complication. The implant was not removed but the patient needed to take antibiotics for recurrent, prolonged periods. Furthermore, several professional oral hygiene treatments had to be performed to reduce the impact of the bacterial infection on the tissues. The incidence of biologic complications was therefore 1.4% (1/67 surviving implants). Four fixed, implant-supported restorations experienced prosthetic problems (fracture of the acrylic implant-supported restoration) during the provisional phase, and in two patients the definitive restoration had ceramic chipping. The incidence of prosthetic complications was 8.9% (6/67 patients). The overall incidence of complications amounted to 14.9% (10/67 patients). All failures and complications encountered during the present study were summarized in Table 1.

## 4. Discussion

Implant-prosthetic treatment in the case of severe maxillary and mandibular bone atrophy has always been a challenge for surgeons [5, 13].

The use of endosseous implants of standard dimension often requires the preliminary use of bone regeneration techniques [4–11, 13]. Such surgical techniques, however, are rather complex, have a high risk of complications, and result in increased time and cost of treatment [5, 6, 10, 13, 15].

In the case of elderly patients with very pronounced bone atrophy, the placement of endosseous implants via bone regenerative techniques may be unwise [13, 42–44]. Such patients may have general (medical) health problems, or may simply not want to pursue a treatment option involving multiple invasive procedures (e.g., hip grafting), the risk of complications and postoperative pain, and increased cost and time of treatment, simply to gain stability and increase masticatory function to that of a complete removable denture [10, 13, 15, 16, 42–44].

Recently, new endosseous implant designs (such as short, narrow, or tilted implants, or zygomatic or pterygomaxillary fixtures) that use the baseline residual bone have been proposed; despite representing valid alternative options, these do require a minimum amount of bone substrate in order to be inserted [16–18] or are surgically demanding [19, 20].

In the case of elderly patients with advanced atrophy, the subperiosteal implant represents another possible alternative [45].

Subperiosteal implants were used widely in the past, before being abandoned in favour of endosseous implants [4, 21–27, 29–31]. The first subperiosteal implant was placed by G. Dahl in 1941 in the lower jaw of a patient in Sweden; Gershkoff and Goldberg were the first to report clinical cases with mandibular subperiosteal implants in the United States [24]. Leonard Linkow is, however, the universally recognized father of subperiosteal implants; in fact, he placed many of these implants and followed them up in multiple clinical studies with follow-up periods ranging from 2 to 12 years [21–24]. Furthermore, Linkow reported on an entirely new mandibular tripodal design concept and made a distinct change to the surgical protocol for obtaining the bone impressions without exposing those parts of the body of the mandible extending from the mental nerves to the ascending rami [21–23].

The popularity of subperiosteal implants declined in the late 1970s due to the rising popularity of the endosseous implants proposed by Branemark [4, 31]. The reasons for the rapid decline of subperiosteal implants included the need for a double intervention, with impressions of the bony bases taken in the first surgical session; the difficulty of manufacturing and positioning during the second surgical session; and the rather high incidence of failures and complications resulting from this [4, 21, 23–30, 33, 35, 46, 47].

In recent years, however, the digital revolution has changed the world of dentistry. New techniques for acquisition and new processing software, combined with the most modern fabrication techniques, have opened up new possibilities, including the customization of implant therapy [36–39].

Today it is possible to perform CBCT and easily obtain complete 3D information on the patient's bone anatomy: we go from the real to the virtual. This information, imported into software dedicated to bone reconstruction, allows us to reconstruct the bone of the patient in 3D. Using the 3D virtual model, it is then possible to design a custom-made implant that is tailored to the specific needs of the patient. Finally, modern digital manufacturing techniques (such as stereolithography) complete the process, transforming the virtual into the real [36–39].

In 1998, McAllister and colleagues reported on the application of stereolithography for the fabrication of a subperiosteal implant [38]. The authors concluded that the advent of stereolithography as a new tool for modeling anatomy for subperiosteal implants and advances in computed tomography offer a higher degree of build accuracy and repeatability than was previously available [38]. Similar results were found by in 2009 by Kusek, who published a case report of a patient who was rehabilitated using a simplified surgical protocol involving laser surgery and stereolithography [39]. In the above papers, the subperiosteal implants were first fabricated by means of CAD/CAM technology (stereolithography) in epoxy resin; then, the resin implants were sent to a dental laboratory for fabrication of the cast frameworks [38, 39].

In the last few years, however, direct metal laser sintering (DMLS) technology has been introduced in the dental field. DMLS is an additive manufacturing (AM) method for creating patterns using thermal fusing (sintering) of powdered metals [40–44, 48]. DMLS models are generated directly from 3D computer data converted to STL files, which are then sliced into thin layers (typically about 0.1 mm/0.004 inches) using appropriate software. The laser sintering machine then produces the models on a moveable platform by applying incremental layers of the pattern metallic material [40–44, 48]. For each layer, the machine lays down a film of powdered metal with a precise thickness (approximately 0.1 mm/0.004 inches) [40–44, 48]. The laser melts selected areas so that they conform to the previous layer. The platform then moves down by the preprogrammed layer thickness, a fresh film of metal powder is laid down, and the next layer is melted via exposure to the laser source. This process continues, layer by layer, until the pattern is completed [44, 48].

DMLS is used today for the fabrication of a range of titanium implants, including endosseous implants of standard sizes [49] as well as custom-made root analogues [42, 43], blades [50], and maxillofacial implants.

Not surprisingly then, the DMLS technique can also be employed for the fabrication of subperiosteal implants of titanium or titanium alloy [44].

In a recently published study, Cohen et al. (2016) reported on new subperiosteal titanium-aluminium-vanadium implants produced by additive manufacturing (AM) and postfabrication osteogenic micro/nanoscale surface texture modification [44]. The authors first studied the* in vitro* biologic behaviour of human osteoblasts when grown on the DMLS surface of these implants and found that the cells produced osteogenic and angiogenic factors, together with a considerable amount of new bone matrix [44]. When implanted in the rat calvaria, these implants had high bone-to-implant contact, with vertical bone growth demonstrated histologically and histomorphometrically [44]. Similar results were found in the tibias of rabbits, but most importantly, new bone formation and excellent osseointegration and bone stability were demonstrated by the same authors in humans* in vivo*, when custom-made subperiosteal DMLS implants were placed to prosthetically rehabilitate the mandibles of two patients [44].

Our present retrospective clinical study seems to confirm these positive outcomes. In fact, in our study of 70 patients who had been treated with custom-made DMLS titanium subperiosteal implants and then followed up for a period of 2 years, a satisfactory implant survival rate (95.8%) was reported, with only three implant failures, due to recurrent, untreatable infection. The immediate postoperative complications were also infrequent, with a low incidence of 5.7%; this low incidence of complications was probably a direct consequence of the reduced surgical time resulting from the excellent fit of custom-made implants on bone sites. Finally, over the 2-year follow-up period, only one patient (1.4%) experienced severe biologic complications; the most frequent complications were technical complications (8.9%) related to the implant-supported fixed restorations.

It must be pointed out that, in our study, the surface of the DMLS titanium subperiosteal implants was polished through electroerosion, so it did not possess the same micro/nanotopographical features of the implants reported by Cohen et al. [44] The possibility of applying a different surface treatment to better exploit the porous surface potential resulting from the DMLS process will certainly be taken into account by our group in the coming years. In fact, excellent histological and histomorphometric findings have been reported in the scientific literature for implants with a direct laser sintered surface [41].

In any case, even without considering the inherent properties of the surfaces of implants fabricated by direct laser manufacturing, the advantages deriving from the application of our new technique for the fabrication of custom-made subperiosteal implants are relevant [44]. First, this new fabrication procedure reduces the number of surgical sessions from two to one, since the direct physical impression of the patient's bone anatomy is no longer needed [44]. This lowers the patient's discomfort and increases compliance with the entire treatment plan, which is shortened in time [44]. Second, all information about the patient's bone anatomy is acquired via CBCT examination and data processing/elaboration using 3D reconstruction software [44]. This means that, besides the exact replica of the bone structure, all relevant anatomic information can be collected before the intervention is performed; such information was not available in the previous conventional protocol [44]. Third, customization makes the fit of the implant more accurate, and the surgical act is therefore easier, faster, and accessible to more surgeons; this may reduce the number of intraoperative complications and the risk of infection [44]. All these benefits have the potential to revitalize the old concept of subperiosteal implants in a modern, digital way [44].

The present study has important limitations, including the short follow-up period, the limited number of patients studied, and the retrospective design. In addition, the placement of subperiosteal implants is rather complex for the clinician and it requires advanced surgical skills, particularly related to the soft tissue management. The same difficulties can be experienced by the clinician, in case of complications: in fact, it may be difficult to treat biologic complications affecting these implants. For all these reasons, medium- and long-term data on a larger sample of patients are necessary before drawing more specific conclusions about the reliability of the present surgical technique, which in any case should only be applied in selected cases (elderly patients with severe bone atrophy who cannot or do not want to undergo complex bone regenerative treatments).

## 5. Conclusions

Within the limits of the present study (limited follow-up time and low number of patients treated, retrospective design), the clinical application of custom-made DMLS titanium subperiosteal implants showed a satisfactory survival rate (95.8%) and low complication rates. In the case of elderly patients with severely atrophic edentulous jaws, the use of custom-made DMLS subperiosteal implants offered the advantages of minimized ridge augmentation treatment needs, reduction in time required to restore lost prosthetic function, and reduced financial burden. Therefore, custom-made DMLS subperiosteal implants may represent a valid alternative treatment procedure for the prosthetic restoration of severely atrophic jaws, where placement of endosseous implants is not possible. However, further studies on a larger sample of patients and with long-term follow-up are needed to confirm the positive outcomes emerging from this clinical research.

## Figures and Tables

**Table 1:** Failure and complications with custom-made subperiosteal implants.

**Gender**	**Age**	**Type of complication**	**Timing of complication**	**Treatment solution**
Male	66	Recurrent untreatable infection	12 months	Implant removal

Male	63	Recurrent untreatable infection	14 months	Implant removal

Female	79	Recurrent untreatable infection	8 months	Implant removal

Female	71	Post-operative pain, discomfort, swelling	1 week	Antibiotic and analgesic therapy
		Fracture of the acrylic temporary prosthetic restoration	2 months	Replacement with a new temporary restoration

Female	72	Post-operative pain, discomfort, swelling	1 week	Antibiotic and analgesic therapy

Male	69	Post-operative pain, discomfort, swelling	1 week	Antibiotic and analgesic therapy

Male	67	Post-operative pain, discomfort, swelling	2 weeks	Antibiotic and analgesic therapy

Female	64	Recurrent infections	20 months	Antibiotic therapy plus professional oral hygiene sessions
		Fracture of the acrylic temporary prosthetic restoration	2 months	Replacement with a new temporary restoration
		Ceramic chipping of the final restoration	7 months	Polishing of the final restoration

Male	71	Fracture of the acrylic temporary prosthetic restoration	3 months	Placement of the final restoration

Female	64	Fracture of the acrylic temporary prosthetic restoration	1 month	Replacement with a new temporary restoration
		Ceramic chipping of the final restoration	16 months	Replacement with a new final restoration

## Data Availability

Data are available upon request, with the consent of both authors.

## References

[1] Mangano C., Mangano F., Shibli J. A., Ricci M., Sammons R. L., Figliuzzi M. (2011). Morse taper connection implants supporting ‘planned’ maxillary and mandibular bar-retained overdentures: a 5-year prospective multicenter study. *Clinical Oral Implants Research*.

[2] Müller F., Al-Nawas B., Storelli S. (2015). Small-diameter titanium grade IV and titanium-zirconium implants in edentulous mandibles: Five-year results from a double-blind, randomized controlled trial. *BMC Oral Health*.

[3] Mangano C., Iaculli F., Piattelli A., Mangano F. (2015). Fixed restorations supported by Morse-taper connection implants: a retrospective clinical study with 10–20 years of follow-up. *Clinical Oral Implants Research*.

[4] Esposito M., Ardebili Y., Worthington H. V. (2014). Interventions for replacing missing teeth: different types of dental implants. *Cochrane Database Systematic Reviews*.

[5] Rocchietta I., Fontana F., Simion M. (2008). Clinical outcomes of vertical bone augmentation to enable dental implant placement: a systematic review. *Journal of Clinical Periodontology*.

[6] Aloy-Prósper A., Peñarrocha-Oltra D., Peñarrocha-Diago M., Peñarrocha-Diago M. (2015). The outcome of intraoral onlay block bone grafts on alveolar ridge augmentations: a systematic review. *Medicina Oral Patología Oral y Cirugía Bucal*.

[7] Felice P., Marchetti C., Iezzi G. (2009). Vertical ridge augmentation of the atrophic posterior mandible with interpositional bloc grafts: Bone from the iliac crest vs. bovine anorganic bone. Clinical and histological results up to one year after loading from a randomized-controlled clinical trial. *Clinical Oral Implants Research*.

[8] Schneider D., Weber F. E., Grunder U., Andreoni C., Burkhardt R., Jung R. E. (2014). A randomized controlled clinical multicenter trial comparing the clinical and histological performance of a new, modified polylactide-co-glycolide acid membrane to an expanded polytetrafluorethylene membrane in guided bone regeneration procedures. *Clinical Oral Implants Research*.

[9] Anitua E., Begoña L., Orive G. (2013). Clinical evaluation of split-crest technique with ultrasonic bone surgery for narrow ridge expansion: Status of soft and hard tissues and implant success. *Clinical Implant Dentistry and Related Research*.

[10] Baas E. M., Van Gemert B. P. H. M., Bierenbroodspot F., Milstein D. M. J., De Lange J. (2015). Patient discomfort and other side effects after bilateral sagittal split osteotomy or distraction osteogenesis of the mandible: a randomized clinical trial. *International Journal of Oral and Maxillofacial Surgery*.

[11] Mangano C., Perrotti V., Shibli J. A. (2013). Maxillary sinus grafting with biphasic calcium phosphate ceramics: clinical and histologic evaluation in man. *The International Journal of Oral & Maxillofacial Implants*.

[12] Mangano C., Sinjari B., Shibli J. A. (2013). A Human clinical, histological, histomorphometrical, and radiographical study on biphasic HA-Beta-TCP 30/70 in maxillary sinus augmentation. *Clinical Implant Dentistry and Related Research*.

[13] Esposito M., Grusovin M. G., Felice P., Karatzopoulos G., Worthington H. V., Coulthard P. (2009). The efficacy of horizontal and vertical bone augmentation procedures for dental implants—a Cochrane systematic review. *European Journal of Oral Implantology*.

[14] Kang Y., Kim H., Byun J. (2015). Stability of simultaneously placed dental implants with autologous bone grafts harvested from the iliac crest or intraoral jaw bone. *BMC Oral Health*.

[15] Barone A., Ricci M., Mangano F., Covani U. (2011). Morbidity associated with iliac crest harvesting in the treatment of maxillary and mandibular atrophies: a 10-year analysis. *Journal of Oral and Maxillofacial Surgery*.

[16] Bechara S., Kubilius R., Veronesi G., Pires J. T., Shibli J. A., Mangano F. G. (2017). Short (6-mm) dental implants versus sinus floor elevation and placement of longer (≥10-mm) dental implants: a randomized controlled trial with a 3-year follow-up. *Clinical Oral Implants Research*.

[17] Mangano F., Shibli J. A., Sammons R. L., Veronesi G., Piattelli A., Mangano C. (2014). Clinical outcome of narrow-diameter (3.3 mm) locking-taper implants: a prospective study with 1 to 10 years of follow-up. *The International Journal of Oral & Maxillofacial Implants*.

[18] Asawa N., Bulbule N., Kakade D., Shah R. (2015). Angulated implants: an alternative to bone augmentation and sinus lift procedure: systematic review. *Journal of Clinical and Diagnostic Research*.

[19] Al-Thobity A. M., Wolfinger G. J., Balshi S. F., Flinton R. J., Balshi T. J. (2014). Zygomatic implants as a rehabilitation approach for a severely deficient maxilla. *The International Journal of Oral & Maxillofacial Implants*.

[20] Balshi T. J., Wolfinger G. J., Slauch R. W., Balshi S. F. (2013). A retrospective comparison of implants in the pterygomaxillary region: Implant placement with two-stage, single-stage, and guided surgery protocols. *The International Journal of Oral & Maxillofacial Implants*.

[21] Linkow L. I., Wagner J. R., Chanavaz M. (1998). Tripodal mandibular subperiosteal implant: basic sciences, operational procedures, and clinical data.. *Journal of Oral Implantology*.

[22] Linkow L. I., Ghalili R. (1999). Ramus hinges for excessive movements of the condyles: a new dimension in mandibular tripodal subperiosteal implants.. *Journal of Oral Implantology*.

[23] Linkow L. I. (2000). Use of a tripodal mandibular subperiosteal implant with ramus hinges for facial asymmetry. *Journal of Oral Implantology*.

[24] Silvestri K. D., Carlotti A. E. (1995). Subperiosteal implant: serving the dental profession for over 50 years.. *Rhode Island dental journal*.

[25] Fettig R. H., Kay J. F. (1994). A seven-year clinical evaluation of soft-tissue effects of hydroxylapatite-coated vs. uncoated subperiosteal implants.. *Journal of Oral Implantology*.

[26] Kurtzman G. M., Schwartz K. (1995). The a viable long-term treatment modality in the severely atrophied mandible: a patient's 40-year case history. *Journal of Oral Implantology*.

[27] Bodine R. L., Yanase R. T., Bodine A. (1996). Forty years of experience with subperiosteal implant dentures in 41 edentulous patients. *The Journal of Prosthetic Dentistry*.

[28] Moore D. J., Hansen P. A. (2004). A descriptive 18-year retrospective review of subperiosteal implants for patients with severely atrophied edentulous mandibles. *The Journal of Prosthetic Dentistry*.

[29] Weiss C. M., Reynolds T. (2000). A collective conference on the utilization of subperiosteal implants in implant dentistry. *Journal of Oral Implantology*.

[30] Schou S., Pallesen L., Hjørting-Hansen E., Pedersen C. S., Fibæk B. (2000). A 41-year history of a mandibular subperiosteal implant. *Clinical Oral Implants Research*.

[31] van Steenberghe D., Brånemark P.-I., Quirynen M., De Mars G., Naert I. (1991). The rehabilitation of oral defects by osseointegrated implants. *Journal of Clinical Periodontology*.

[32] Linkow L. I., Ghalili R. (1998). Critical design errors in maxillary subperiosteal implants.. *Journal of Oral Implantology*.

[33] Sconzo J. (1998). The complete mandibular subperiosteal implant: an overview of its evolution.. *Journal of Oral Implantology*.

[34] Golec T. S., Krauser J. T. (1992). Long-term retrospective studies on hydroxyapatite coated endosteal and subperiosteal implants.. *Dental Clinics of North America*.

[35] Benson D., Reisbick M. H., Furstman L. L. (1974). Ceramic-coated subperiosteal implants. Part II. Clinical and histologic evaluations. *The Journal of Prosthetic Dentistry*.

[36] van Noort R. (2012). The future of dental devices is digital. *Dental Materials*.

[37] Colombo M., Mangano C., Mijiritsky E., Krebs M., Hauschild U., Fortin T. (2017). Clinical applications and effectiveness of guided implant surgery: A critical review based on randomized controlled trials. *BMC Oral Health*.

[38] Imburgia M., Logozzo S., Hauschild U., Veronesi G., Mangano C., Mangano F. G. (2017). Accuracy of four intraoral scanners in oral implantology: a comparative in vitro study. *BMC Oral Health*.

[39] Joda T., Zarone F., Ferrari M. (2017). The complete digital workflow in fixed prosthodontics: a systematic review. *BMC Oral Health*.

[40] Sonmez N., Gultekin P., Turp V., Akgungor G., Sen D., Mijiritsky E. (2018). Evaluation of five CAD/CAM materials by microstructural characterization and mechanical tests: a comparative in vitro study. *BMC Oral Health*.

[41] Ciocca L., Fantini M., De Crescenzio F., Corinaldesi G., Scotti R. (2011). Direct metal laser sintering (DMLS) of a customized titanium mesh for prosthetically guided bone regeneration of atrophic maxillary arches. *Medical & Biological Engineering & Computing*.

[42] Mangano F. G., de Franco M., Caprioglio A., Macchi A., Piattelli A., Mangano C. (2014). Immediate, non-submerged, root-analogue direct laser metal sintering (DLMS) implants: a 1-year prospective study on 15 patients. *Lasers in Medical Science*.

[43] Mangano F. G., Cirotti B., Sammons R. L., Mangano C. (2012). Custom-made, root-analogue direct laser metal forming implant: a case report. *Lasers in Medical Science*.

[44] Cohen D. J., Cheng A., Kahn A. (2016). Novel osteogenic Ti-6Al-4V device for restoration of dental function in patients with large bone deficiencies: design, development and implementation. *Scientific Reports*.

[45] Nazarian A. (2014). Placement of a modified subperiosteal implant: a clinical solution to help those with no bone.. *Dentistry Today*.

[46] Mansueto R. F. (1999). Replacement of a mandibular subperiosteal implant.. *Journal of Oral Implantology*.

[47] Markiewicz M. R., Nishiyama K., Yago K. (2007). Draining orocutaneous fistula associated with a failing subperiosteal implant: report of a case.. *Journal of Oral Implantology*.

[48] Traini T., Mangano C., Sammons R. L., Mangano F., Macchi A., Piattelli A. (2008). Direct laser metal sintering as a new approach to fabrication of an isoelastic functionally graded material for manufacture of porous titanium dental implants. *Dental Materials*.

[49] Mangano C., Mangano F., Shibli J. A. (2012). Prospective clinical evaluation of 201 direct laser metal forming implants: results from a 1-year multicenter study. *Lasers in Medical Science*.

[50] Mangano F., Bazzoli M., Tettamanti L. (2013). Custom-made, selective laser sintering (SLS) blade implants as a non-conventional solution for the prosthetic rehabilitation of extremely atrophied posterior mandible. *Lasers in Medical Science*.

